# Method of evaluating the impact of landfill leachate on groundwater quality

**DOI:** 10.1007/s10661-018-6776-2

**Published:** 2018-06-20

**Authors:** Kazimierz Szymański, Beata Janowska, Anna Iżewska, Robert Sidełko, Izabela Siebielska

**Affiliations:** 1 Faculty of Civil Engineering, Environmental and Geodetic Sciences, Department Waste Management, Koszalin University of Technology, ul. Śniadeckich 2, 75-453 Koszalin, Poland; 2 Faculty of Civil Engineering and Architecture, Department of Sanitary Engineering, West Pomeranian University of Technology Szczecin, Al. Piastów 50, 70-311 Szczecin, Poland

**Keywords:** Landfill leachate, Aeration zone, Water pollution, Migration

## Abstract

Closed municipal and industrial waste landfill sites create potential hazard of ground water pollution. Pollutants that occur in leachate infiltrate to the soil substratum, where they are carried to in underground water. A municipal waste landfill substratum can be used for elimination of pollutants contained in leachates. Model research was performed with the use of a sand bed and artificially prepared leachates. Efficiency of filtration in a bed of defined thickness was assessed based on change of COD value. Results of the model tests have indicated that the mass of pollutants contained in leachate filtered through porous ground layer depends on the mass of supplied pollutants, intensity of supplied leachate, and layer thickness. Increase of the mass of pollutants supplied to a unit area of ground layer causes reduction of the relative value of COD mass. The method of evaluation of quality of water seeping through the aeration layer presented in this paper allows for estimation of the flowing out pollutants mass. Based on the test results obtained, efficiency of purification in the aeration zone can be assessed; likewise, safe thickness of the filtration layer under the landfill site can be designed.

## Introduction

Municipal waste landfill sites, which are placed on permeable soil, create potential hazard for purity of ground waters located in their neighbourhood (Szymański et al. 2007). Type and quantity of pollutants migrating from any landfill site to ground waters depends mainly on the type of landfilled waste, its quantity, site’s geometry, physical condition of the substratum, and thickness of the aeration zone (Han et al. 2013; Li et al. 2012; Nowak et al. 2016; Regadio et al. 2012; Reyes-López et al. 2008). Mass of the pollutants migrating from the aeration zone to ground waters can be estimated by application of various methods (Koda et al. 2009; Szymański and Siebielska 2000; Wysocka 2015). The best possible case takes into consideration observations performed on real objects. Such methods are usually time-consuming and expensive, particularly if they are applied in closed objects. The degree of pollution of underground waters can be estimated based on model test results (Burn and Engesgaard 2002; Thornton et al. 2005). Model tests should be performed taking into account the conditions of landfill location, qualitative and quantitative parameters of landfill leachate, and underground water (Szymański and Janowska 2016; Tsanis 2006; Tałałaj and Dzienis 2007; Zhu et al. 2013). An attempt was made in this paper to estimate migration of leachate to the aeration zone based on artificially modelled soil substratum and artificial landfill leachate.

## Theoretical basis for the process of migration of leachate to ground waters

**Fig. 1:**
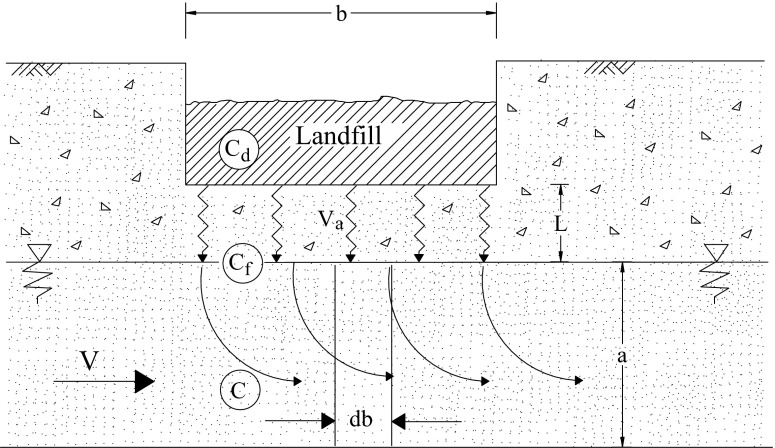
Diagram of pollutants inflowing into underground waters (aeration zone) (Szymański 1987)

Methods of determination of pollutant concentrations in ground waters flowing in the water-bearing layer featuring thickness *a*, with velocity v, under a landfill featuring width *b* (Fig. 1), were described in many publications (Burn and Engesgaard 2002; Islam and Singhal 2004; Lacerda et al. 2014; Li et al. 2012; Nayak et al. 2007; Regadio et al. 2012; Tsanis 2006). Such calculations are based on determination of elementary volumes of leachate filtrated through the aeration zone *dV*_*f*_, flowing within period *dt*, into ground water surface *db* of 1.0 m^2^_._ In such case, the following formula can be used (1):

1 $$ d{V}_f={v}_a\bullet db\bullet 1.0\bullet dt $$

where *v*_a_ means velocity of leachate flow in the aeration zone.

Total volume of leachate, which flows into that area, during its flow with velocity **v**, at the distance equal to the landfill site width, can be determined from the following formula (2):

2 $$ {V}_f={v}_a\bullet {\int}_0^b db\bullet {\int}_0^T dt={v}_a\bullet b\bullet T $$

Time ***T*** is equivalent to a period in which front of the considered ground water surface will move by distance ***b***. This can be calculated from the following formula:

3 $$ T=\frac{b}{v} $$

therefore, volume of leachate arriving to a single square meter of water surface will be equal to:

4 $$ {V}_f^{\prime }=\frac{v_a}{v}\bullet b $$

Therefore, concentration of pollutants in the ground water flowing out of the landfill site area (without taking into account of self-purification of those waters during their flow under the landfill site) can be calculated from the following formula:

5 $$ C=\frac{c_f\bullet {v}_f^{\prime }}{a}={C}_f\frac{v_a\bullet b}{v\bullet a} $$

where **C**_**f**_ is a concentration of any pollution indicator in water flowing out from the aeration zone.

The approximate liquid flow velocity in the aeration zone ***v***_***a***_ can be calculated from a formula proposed by Szestakow (1973):

6 $$ {v}_a=\frac{1}{n_0}\bullet {\omega}^{\left(1-\frac{1}{n}\right)}\bullet {k}^{\frac{1}{n}} $$

where particular symbols define:

n_o_: active soil porosity,
intensity of leachate outflow from landfill,
k: coefficient of permeability of the aeration zone soil,
n: a coefficient, which in this calculation assumes value of *n* = 3.

Therefore, the approximate leachate velocity value in the aeration zone is

7 $$ {v}_a=\frac{1}{n_0}\bullet \sqrt[3]{\omega^2\bullet k} $$

Analysis of formula (5) leads to an observation that concentration of pollutants in ground water increases with increase of concentration of inflowing leachate (C_f_), velocity of its flow in the aeration zone and landfill size. An assumption has been made that landfill leachate migrates in the same direction as ground waters flow. Underground water pollutant concentration is contingent also upon conditions of their flow. The lower the volumetric output of those waters, the higher the probability of their pollution.

**Fig. 2:**
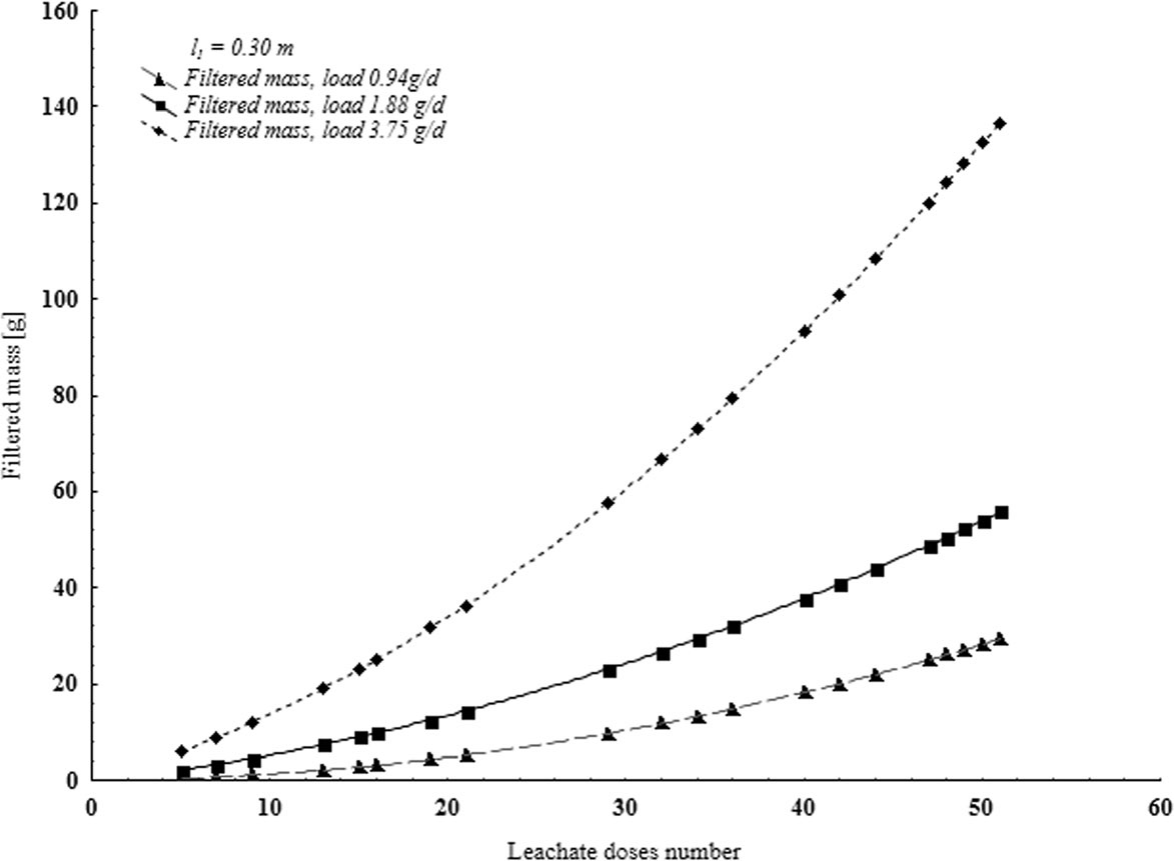
Pollutant load contained in the filtrate after flowing through 0.30 m bed expressed by COD K_2_Cr_2_O_7_

**Fig. 3:**
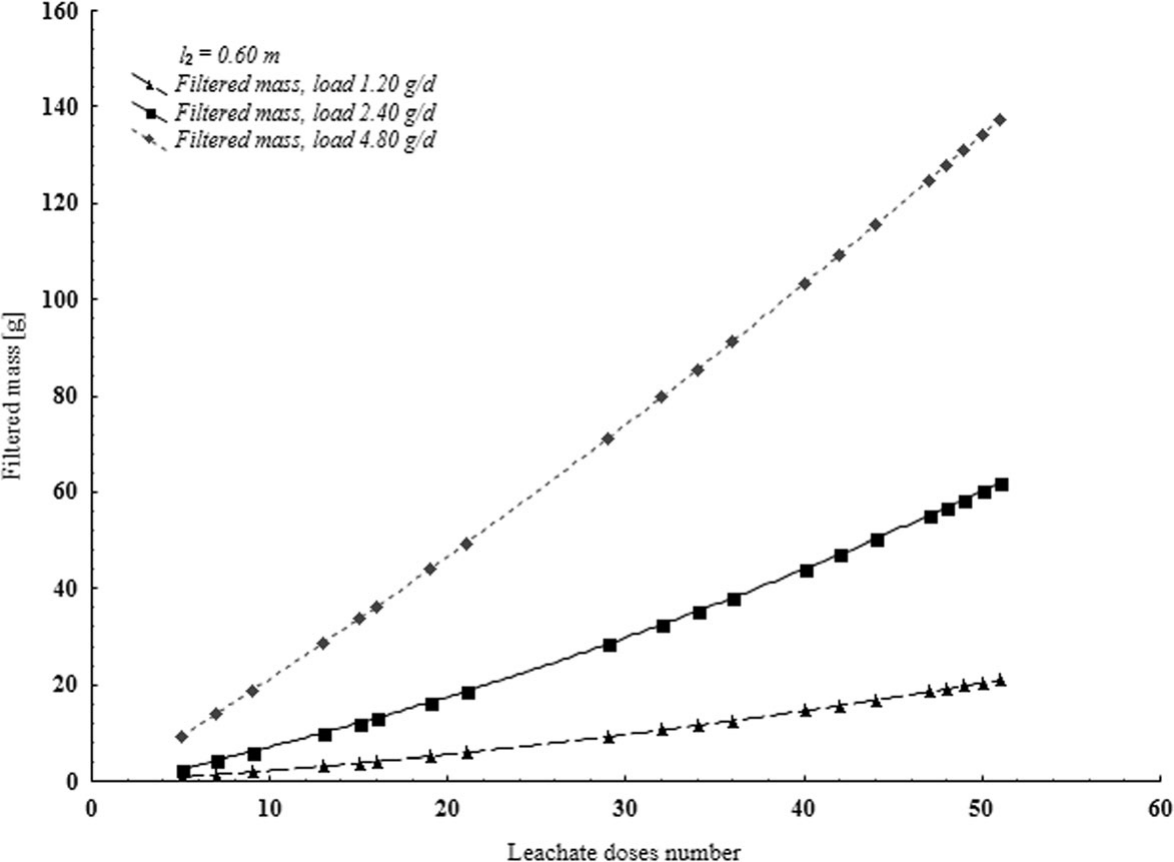
Pollutant load contained in the filtrate after flowing through 0.60 m bed expressed by COD K_2_Cr_2_O_7_

**Fig. 4:**
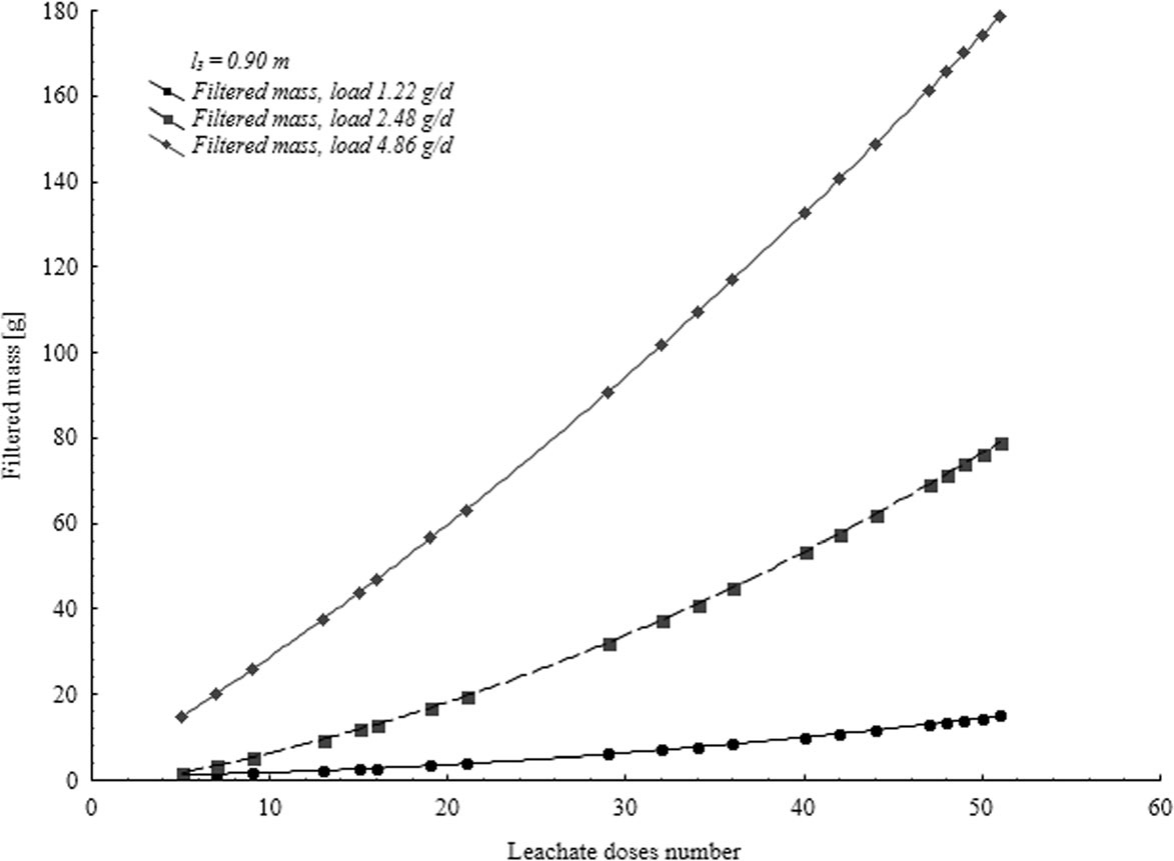
Pollutant load contained in the filtrate after flowing through 0.90 m bed expressed by COD K_2_Cr_2_O_7_

Concentration of pollution in ground water flowing outside the landfill perimeter can be calculated much easier if test results for aeration zone purification properties (Figs. 2, 3, and 4) are available:

8 $$ Q=\frac{m_d}{F\bullet t}=\frac{m_d^{\prime }}{t} $$

where particular symbols define:

m_d_: mass of supplied pollutants
m^’^_d_: mass of pollutant supplied into soil unit area.

Assuming permanent load of supplied leachate *(Q)*, mass of pollutant supplied to a single square meter of the aeration zone *F* (sand layer) can be calculated for any period *t*:

9 $$ {\left({m}_d^{\prime}\right)}_t=Q\bullet t $$

Based on known ground water flow time *T* at distance *b*, mass of supplied pollutants (in period *t + T*) can be determined from the following formula:

10 $$ {\left({m}_d^{\hbox{'}}\right)}_{t+T}=Q\left(t+T\right) $$

Using model test results, as a relationship between *m*_*f*_^*’*^ (mass of pollutant filtered through unit area) and ***m***_*d*_^'^, mass of pollutants inflowing to ground water during their flow under the landfill *(m*_*f*_^*’*^*)*_*T*_ can be determined. Therefore, concentration of pollutants in water flowing beyond the landfill perimeter will be equal to:

11 $$ C=\frac{{\left({m}_f^{\hbox{'}}\right)}_T}{a} $$

If a linear relation would occur between *m*_*f*_^*’*^ and *m*_*d*_^*’*^ values, then, values of *(m*_*f*_^*’*^*)*_*T*_ will be constant and independent of time. In the event that the relationship between those values would be described by another function, values *(m*_*f*_^*’*^*)*_*T*_ will change in time of landfill operation period **(*t*)**. In such case, this variable should be taken into account in calculation of pollutant concentration in ground water flowing out of the considered object area.

## Materials and methods

Model tests representing infiltration of leachate through the aeration zone were performed in order to define the qualitative and quantitative relationship between the mass of pollutants both supplied and carried away from the porous medium (An et al. 2013; Lacerda et al. 2014; Papapertridis and Paleologos 2012; Szymański and Janowska 2016). Medium sand and artificially prepared leachates were used to fill in the filtration columns. The physico-chemical composition of the leachates was selected in such way so as to reflect content of selected indicators in real leachates (Castrillon et al. 2010; Szymański and Nowak 2012). The variable parameters were intensity of supplied leachates ***ω*** (leachate volume per unit area and unit of time), mass of supplied pollutants *m*_*d*_, and soil thickness *l*. Analysis of changes in the mass of pollutants *m*_*f*_ filtered through the model layer was performed using one of the indicators describing landfill leachate such as COD K_2_Cr_2_O_7_ (Kulikowska and Klimiuk 2008).

Medium sand taken from the municipal landfill area was used in the tests.

Its maximum and minimum density values were *(ρ*_*d*_*)*_max_ = 1.79∙10^3^kg∙m^−3^ and *(ρ*_*d*_*)*_min_ = 1.63∙10^3^kg∙m^−3^ respectively. The maximum sand density at humidity *w* = 2.3%, in which it was used in model tests, was *ρ*_max_ = 1.83∙10^3^ kg m^−3^. Approximately 2.0 kg of sand was being poured into the column and then compacted with 1.0 kg compactor, which was dropped ten times from the height of approximately 0.5 m. Once the bed was formed up to a defined height, its depth was measured and density was calculated (Szyszkowski, 2000). Three series of tests were performed. Each series differed in bed thickness, which was *l*_1_ = 0.3 m, *l*_*2*_ = 0.6 m, *l*_*3*_ = 0.9 m respectively. In each test, series leachate was supplied everyday into respective columns with variable intensity: ω_1_ = 0.026 m^3^∙m^−2^∙d^−1^, ω_2_ = 0.052 m^3^∙m^−2^∙d^−1^, ω_3_ = 0.104 m^3^∙m^−2^∙d^−1^, and (ω_2_ = 2∙ω_1_, ω_3_ = 4∙ω_1_). Each day a specified dose of leachate was supplied to the top surface of the filter bed. Therefore, to the first filtration column, in each test series, following volumes of leachate were supplied: *v*_1_ = 195∙10^−6^ m^3^, to the second *v*_*2*_ = 390∙10^−6^ m^3^, and to the third *v*_*3*_ = 780∙10^−6^ m^3^ respectively. During the entire test procedure, approximately 50 doses of leachate were supplied to each column. COD K_2_Cr_2_O_7_ values were then determined in the leachates and filtrates (Szyszkowski, 2000).

## Results and discussion

Values of pollutant loads in particular experiments are given based on COD example (Figs. 2, 3, and 4). Figures 2, 3, and 4 show mass of pollutants contained in the filtrate depending on the number of supplied doses of leachate for all three series of tests. To each layer of given series (*l* = const), various volumes of leachate were supplied (*V*_*1*_, *V*_*2*_, or *V*_*3*_), so intensity (*ω*_*1*_*, ω*_*2*_ or *ω*_*3*_) and mass of supplied pollutants *(m*_*d*_*)* was different. Mass of pollutants in the filtrate depends on thickness of the bed and intensity of leachate supplied.

In each test series, there was a different value of pollutant mass in a dose of supplied leachate. Pollutant load shows, depending on increase of leachate volume and intensity, a growing trend of the mass of filtered pollutants.

Increasing of volume and decreasing of leachate supply intensity extends the test time considerably. Constant increase of the mass of filtered pollutants (Figs. 2, 3, and 4, Table 1) was observed for certain values of supplied leachate ***m***_***d***_. In the final stage of the experiments, ∆m_f_/∆m_d_ ratio assumed constant values. Therefore, relationship obtained for slightly higher ***m***_***d***_ values than those applied in the tests can be extrapolated. One can expect that if the layer thickness will tend to zero, mass of filtrated pollutants will approach the mass of pollutants supplied. However, if intensity of supplied leachate will approach zero, also mass of the filtered impurities will approach zero. Assuming correctness of this reasoning, the following assumptions were made:

$$ {\displaystyle \begin{array}{cc} if\kern0.5em I=0\kern0.5em to\kern0.5em {m}_f={m}_d& \left( if\kern0.5em y=0\kern0.5em to\kern0.5em Z=x\right)\\ {} if\kern0.5em \omega =0\kern0.5em to\kern0.5em {m}_f=0& \left( if\kern0.5em w=0\kern0.5em to\kern0.5em Z=0\right)\end{array}} $$

where ***Z***—regression function coefficients calculated for the relationship between the mass of pollutants supplied and filtered through the layer of medium sand (Table 2). Therefore, function *Z* = *Z*(*x*, *y*, *w*) can be expressed in the following way:

**Table 1:** Regression function coefficients calculated for the relationship between mass of pollutants supplied and filtered through the medium sand bed (*F* = 0.0075 m^2^)

Pollution	Thickness	Intensity	*Z* = a x^b^			*Z* = a x + b			*Z* = a x^2^ + bx + c			
Indicator	[m]	[m^3^∙m^−2^∙day^−1^]	(m_f_ = a m_d_^b^)			(m_f_ = a m_d_ + b)			(m_f_ = a m_d_^2^ + b m_d_ + c)			
			*a*	*b*	*R* ^2^	*a*	*b*	*R* ^2^	*a*	*b*	*c*	*R* ^2^
1	2	3	4	5	6	7	8	9	10	11	12	13
COD	0.3	0.026	0.042	1.63	0.925	0.46	− 2.77	0.978	0.009	0.049	0.755	0.996
		0.052	0.043	1.6	0.982	0.57	− 5.38	0.992	0.003	0.269	− 0.629	0.999
		0.104	0.061	1.48	0.993	0.69	− 11.02	0.995	0.002	0.409	− 2.958	0.999
	0.6	0.026	0.119	1.22	0.942	0.38	− 4.07	0.971	0.006	− 0.068	2.967	0.999
		0.052	0.06	1.45	0.007	0.59	− 11.02	0.997	0.001	0.426	− 5.834	0.999
		0.104	0.102	1.32	0.998	0.67	− 20.49	0.997	0.001	0.452	− 6.601	0.999
	0.9	0.026	0.039	1.3	0.852	0.2	− 2.39	0.946	0.005	− 0.13	1.748	0.998
		0.052	0.103	1.34	0.979	0.62	− 9.48	0.992	0.002	0.353	− 3.162	0.995
		0.104	0.344	1.23	0.997	0.69	− 6.61	0.998	0.001	0.738	− 8.995	0.998

**Table 2:** Linear function of multiple regression for selected indicator describing leachate supplied to an exemplary filtration bed featuring thickness of 0.90 m

Pollution indicator	Regression function m_f_^’^ = m_f_^’^ (m_d_, *l*, ω)	Multiple correlation coefficient *R*^2^
COD K_2_Cr_2_O_7_	m_f_^’^ = 0.54 m_d_^’^ + 291.751 + 25,485.90 ω – 2162.76	0.9957

m_f_^’^—mass of pollutant filtered through unit area [g∙m^−2^], m_d_^’^—mass of pollutant supplied into soil unit area [g∙m^−2^], *l*—layer thickness [m], ω—intensity of leachate supply [m^3^∙m^−2^ day^−1^]

12 $$ Z\left(x,y,w\right)=w\left[{Z}_1^{\prime}\left(x,y\right)\bullet {K}_1^{\prime }+{Z}_2^{\prime}\left(x,y\right)\bullet {K}_2^{\prime }+{Z}_3^{\prime}\left(x,y\right)\bullet {K}_3^{\prime}\right] $$

***Z*** function values are taken for given pollution indicator, depending on layer thickness *y* and intensity *w*, from Table 2. The parameters of regression function presented in said table were calculated for laboratory conditions in which leachate flew through area of *F* = 0.0075 m^2^. In calculations performed for 1.0 m^2^, area those parameters should be corrected. If linear relationship will be adopted (in the case of COD) between the mass supplied and filtered, then coefficient *a* will not change whereas coefficient *b’* at the free term can be calculated from the following formula:

13 $$ {b}^{\prime }=b\bullet F=b\bullet 0.0075 $$

Taking into consideration the influence of all three variables (m_d_, *l*, ω), formula (12) will take the following form:

14 $$ Z=0.0034\left[7.413\bullet 92.61+10.882\bullet \left(-32.27\right)+12.677\bullet 2.60\right] $$

Finally, COD mass inflowing during a single day to ground water from 1.0 m^2^ of the landfill will be

$$ \Delta  Z={\left(\Delta  {m}_f^{\prime}\right)}_{COD}=1.252\frac{g}{m^2}d. $$

Determined regression functions indicate compatibility with linear model of empiric values of variable *m*_*f*_^*’*^${\mathrm{m}}_{\mathrm{f}}^{\prime }$. Determined regression functions allow for estimation of qualitative and quantitative influence of analysed independent variables ***(***${m}_d^{\prime }$*, l, ω)* onto values of the mass of pollutants flowing out from the medium sand layer. The presented model can only be used to estimate leaching of contaminants from landfills if the landfill subsoil is made of sandy soil. These model have been verified in the studies, which were presented by Szymański and Janowska (2016) and Szymański et al. (2017).

## Conclusion

The proposed method of estimation of mass of pollutants penetrating through the aeration zone to ground waters can make the first approximation in solution of practical problems (Islam and Singhal 2004; Nayak et al. 2007). However, this method does not allow for direct identification of the processes that occur during flow of polluted water through a porous medium. Yet, use of the real soil in model tests (taken from the substratum of the tested landfill) and representative composition of a leachate, allows for obtaining reliable information on pollution of ground water in the considered specific case (An et al. 2013; Cuevas et al. 2012). The method of assessment of quality of water seeping through the aeration layer presented in this paper allows for estimation of the mass of outflowing pollutants and intensity of supply of pollutants to the saturation layer.

Based on the test results obtained, efficiency of purification of the aeration zone can be assessed. Safe thickness of the filtration layer under the landfill can also be designed. At the same time, a forecast of the speed of propagation of the pollution zone in ground water and the distance of such zone front from the landfill in various periods of its operation can be forecast. The model test results for migration of landfill leachate in soil substratum, interpreted by application of the linear function of multiple regression, indicate a possibility of their adaptation to specific soil conditions (Szymański and Janowska 2016). They allow also for forecasting of the types of pollution that could occur in landfill leachate infiltrating to the substratum composed of porous materials and to underground waters.
